# The Principles and Practice of Disinfection

**Published:** 1867-09

**Authors:** 


The Principles and Practice of Disinfection. By ROBERTS BARTHOLEW, A.M., M.D., Professor Materia Medica and Therapeutics in the Medical College of Ohio, etc., etc., etc. Cincinnati: R. W. CARROLL & Co., West 4th Street. 1867.

This is a very neatly printed monograph of 111 pages. It may be read with pleasure and profit by all who are interested in the important subject of disinfection.

